# Enhancing Ultrasonic Crack Sizing Accuracy in Rails: The Role of Effective Velocity and Hilbert Envelope Extraction

**DOI:** 10.3390/mi17030346

**Published:** 2026-03-12

**Authors:** Trung Thanh Ho, Toan Thanh Dao

**Affiliations:** Department of Electronic Engineering, University of Transport and Communications, No. 3, Cau Giay Street, Hanoi 100000, Vietnam; trunght@utc.edu.vn

**Keywords:** ultrasonic testing, crack depth estimation, Hilbert transform, effective velocity, digital signal processing, non-destructive testing

## Abstract

Ultrasonic testing is a prevalent method for non-destructive evaluation of railway rails; however, conventional Time-of-Flight (*ToF*) approaches applied in practical dry-coupled inspections often rely on simplified assumptions regarding wave propagation velocity and neglect complex waveform characteristics. This paper presents a robust depth estimation framework for surface-breaking cracks that enhances sizing accuracy through effective velocity calibration and Hilbert envelope extraction. Unlike standard methods that assume the free-space speed of sound in air (343 m/s) for wave propagation within the air-filled gap of a surface-breaking crack, we propose an effective velocity model derived from in situ calibration to account for the boundary layer viscosity and thermal conduction effects within narrow crack geometries. The signal processing chain incorporates spectral analysis, band-pass filtering, and Hilbert Transform-based envelope detection to mitigate noise and resolve phase ambiguities. Experimental validation on steel specimens with controlled defects (0.2–10.0 mm) demonstrates that the proposed method achieves an exceptional linear correlation (*R*^2^ ≈ 0.9976). The calibrated effective velocity was determined to be 289.3 m/s, approximately 15.6% lower than the speed of sound in air, confirming the significant influence of confinement effects. Furthermore, excitation parameters were optimized, identifying that high-voltage excitation (≥110 V) and a tuned pulse width (≈150 ns) are critical for maximizing the signal-to-noise ratio. The results confirm that combining physical model calibration with advanced signal analysis significantly reduces systematic errors, paving the way for portable, high-precision rail inspection systems.

## 1. Introduction

The rapid development of high-speed railway systems has significantly reshaped modern land transportation by enabling fast, safe, and energy-efficient mobility. Countries such as Japan, China, European nations, and several Asian countries including Vietnam have established extensive high-speed rail networks, imposing increasingly stringent requirements on infrastructure reliability and inspection quality [1]. As operating speed and axle load increase, rail components are subjected to higher mechanical and thermal stresses, making structural integrity monitoring a critical aspect of maintenance strategies [2]. During long-term service, rails experience complex loading conditions involving rolling contact fatigue and environmental influences, leading to the initiation of surface and subsurface defects such as head checks, squats, and transverse cracks [2,3,4]. The progressive nature of these defects highlights the necessity of non-destructive testing (*NDT*) techniques capable of detecting early-stage damage with high sensitivity.

Among various *NDT* approaches, ultrasonic testing remains the most widely adopted technique for rail inspection due to its ability to detect internal discontinuities [2,5]. Conventional ultrasonic inspection relies on transmitting acoustic pulses and analyzing reflected signals. Recently, significant attention has been given to Time-of-Flight (ToF)-based approaches, where defect depth is inferred from the wave propagation time. Rather than a singular methodology, these approaches encompass a diverse spectrum of techniques. This includes broad structural evaluations [6,7,8], advanced non-contact laser systems [9], theoretical elasticity models [10], and various surface or guided-wave methods [11,12,13,14,15]. Furthermore, specific advancements in ToF signal processing have progressively improved measurement precision. This evolution spans from peak tracking models and acoustic ToF algorithms [16,17,18], through foundational elastic wave theories [19,20], to specialized methods addressing zero-crossing and material attenuation [21,22,23,24]. More critically for the context of this study, our proposed framework is directly founded on robust analytical envelope detection [25] and optimized pulse excitation techniques [26]. However, conventional ToF methods often rely on simplified linear assumptions, treating wave propagation in confined cracks as identical to free-space transmission. This simplification neglects the complex interaction between the acoustic wave and the crack boundaries, leading to systematic errors in depth estimation.

Several studies have investigated advanced ultrasonic techniques to address these challenges. Pathak et al. [9] and Lian et al. [11] developed laser-induced and guided-wave systems for detecting flaws in rail feet and surfaces. While effective, these non-contact methods often require complex optical setups and suffer from lower signal-to-noise ratios (*SNRs*) compared to contact methods. To improve timing accuracy, signal processing algorithms have also evolved. Yang et al. [16] introduced peak tracking models, and Tumšys [22] proposed zero-crossing methods for filtered signals. However, zero-crossing techniques remain sensitive to phase dispersion caused by material attenuation, potentially leading to “cycle-skipping” errors. Furthermore, while Wang et al. [26] demonstrated that composite (coded) pulse excitation can enhance *SNR* in attenuative media, such techniques require complex correlation processing hardware, which limits their applicability in low-cost, portable devices.

A critical gap remains in the literature regarding the rigorous calibration of propagation velocity in narrow defects. Most existing methods, such as the air-coupled approach by Bühling et al. [21], assume the speed of sound in air (*c*_air_ ≈ 343 m/s) for surface-opening cracks, disregarding the viscous drag and thermal conduction effects imposed by the steel walls. Furthermore, relying solely on time-domain thresholding without analyzing the spectral content and energy envelope can result in significant inaccuracies due to phase ambiguity and structural noise [25].

In this work, we propose an enhanced ultrasonic depth estimation framework that integrates a physically calibrated effective velocity model with advanced digital signal processing (*DSP*). Unlike traditional approaches, this study explicitly addresses the frequency-dependent attenuation and boundary confinement effects in rail steel. The methodology incorporates spectral analysis to identify the resonant response, Hilbert Transform for precise phase-independent envelope detection, and an in situ calibration of the effective propagation velocity (*v*_eff_) to minimize systematic errors. Additionally, we perform a systematic optimization of excitation parameters (voltage and pulse width) to demonstrate that a simple, tuned high-voltage pulse can achieve high precision without the need for complex coded excitation. This combined approach aims to provide a robust solution for rail defect characterization, suitable for future implementation in portable inspection systems.

## 2. Experimental Methods

**Figure 1:**
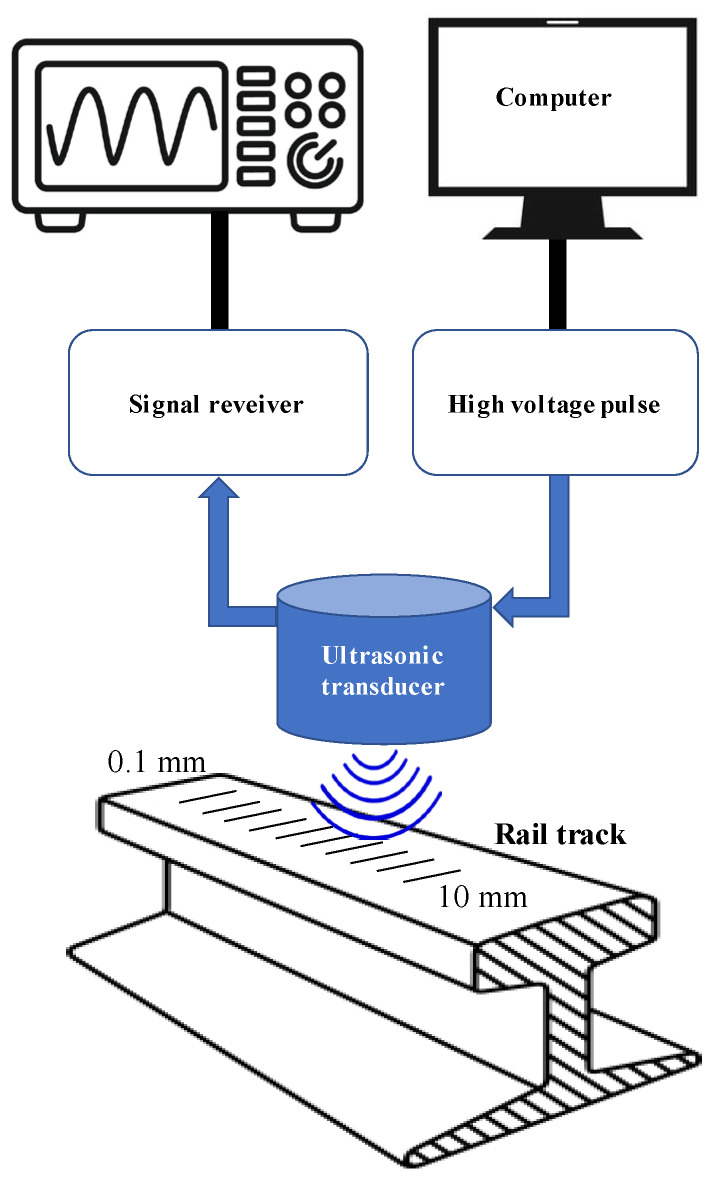
System design of ultrasonic non-destructive testing system for rail track inspection.

**Figure 2:**
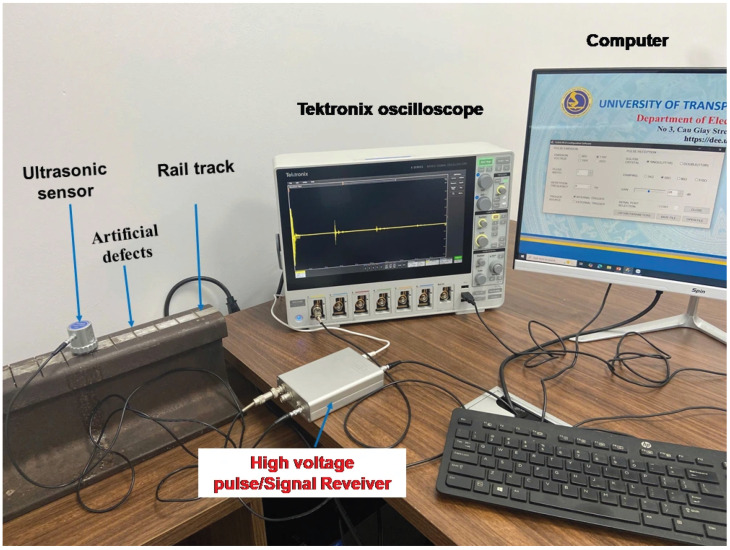
Photo of completed ultrasonic non-destructive testing system for rail track inspection.

The ultrasonic non-destructive testing framework developed in this study is conceptually illustrated in Figure 1 (system architecture) and physically implemented as shown in Figure 2. The measurement system comprises four principal modules: a broadband ultrasonic transducer, a pulse generation/reception unit, a high-fidelity digitization interface, and a host computer for digital signal processing. A piezoelectric ultrasonic transducer with a nominal center frequency of 4 MHz (Yushi Instruments, Shenyang, China) was employed in a pulse-echo configuration, acting alternately as the transmitter and receiver. This nominal frequency was selected to balance the trade-off between penetration power and axial resolution for detecting shallow defects. The inspection uses a contact-based pulse-echo transducer acting directly on the steel surface with an acoustic coupling medium, and the air velocity calibration applies strictly to the wave phase traversing the crack’s physical gap. The excitation is managed by a PR10 pulser/receiver unit (Yushi Instruments), which generates high-voltage negative square pulses to drive the transducer and subsequently amplifies the backscattered echoes. The pulse duration is actively controllable via the pulser unit and was nominally set to 150 ns for the baseline measurements—a value selected based on subsequent parameter optimization analysis. The validation specimen used in the experiments is a representative section of high-carbon steel rail utilized in the Ben Thanh-Suoi Tien metro line (Ho Chi Minh City, Vietnam). The specimen was mechanically stabilized on a laboratory bench to eliminate vibrational noise. To simulate varying degrees of structural damage and, crucially, to serve as a calibration standard for the effective velocity model, a series of artificial cracks were machined into the rail surface. While artificial transverse cracks are used for foundational calibration, real-world rail defects often present complex geometries (e.g., angled or closed), which will necessitate further model expansion in future field studies. These defects consist of narrow transverse grooves with precisely controlled depths ranging from 0.1 mm to 10.0 mm, and a uniform nominal gap width of 0.5 mm, covering the spectrum from incipient micro-cracks to severe structural flaws [3]. All experiments were conducted under controlled laboratory conditions at room temperature, with an acoustic coupling medium applied between the transducer and the rail surface to minimize impedance mismatch.

To ensure high-fidelity data capture for verifying the proposed signal processing algorithms, specifically the FFT (Fast Fourier Transform) and Hilbert Transform validation, the analog output from the pulser/receiver was digitized using a Tektronix MSO46B digital oscilloscope (Tektronix, Beaverton, OR, USA). The signals were sampled at a high rate of 1.25 GS/s to preserve the fine waveform details and phase information required for accurate ToF analysis. While this high-precision oscilloscope was utilized for the validation phase in this study, the proposed methodology is designed to be compatible with standard embedded hardware for future portable field inspections. The digitized time-domain waveforms were synchronized with the excitation pulse and transferred to a computer for the offline processing steps detailed in the subsequent section.

## 3. Results and Discussion

### 3.1. Waveform Analysis and Signal Processing Efficacy

**Figure 3:**
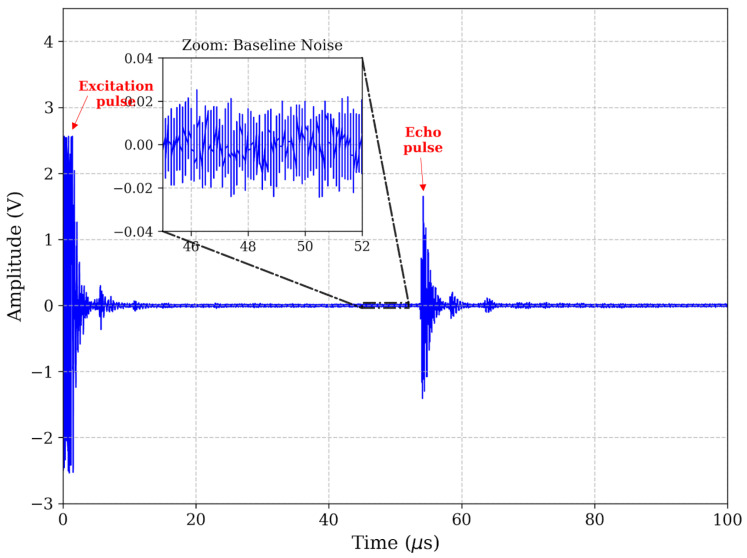
Raw ultrasonic signal in time domain.

The accuracy of ToF estimation in ultrasonic non-destructive testing depends fundamentally on the fidelity of the received transient response. In this study, the efficacy of the proposed digital signal processing chain was evaluated by analyzing the ultrasonic echo returned from a representative 8 mm depth defect (*d*). As shown in Figure 3, the raw time-domain signal (*s*_raw_(t)) exhibits substantial baseline drift and superimposed broadband noise, which are typical challenges in practical dry-coupled inspections. While the primary defect echo is discernible, the low *SNR* renders standard threshold-crossing methods unreliable, as the triggering point becomes highly susceptible to amplitude fluctuations.

To elucidate the spectral characteristics of these distortions, an *FFT* (Fast Fourier Transform) was performed on the raw signal, defined as

(1) $$X\left(f\right)=\sum_{n=0}^{N-1}s_{raw}\left[n\right]e^{-j2\pi fn/N}$$

where *N* represents the number of samples and *s_raw_* [*n*] is the discrete signal.

**Figure 4:**
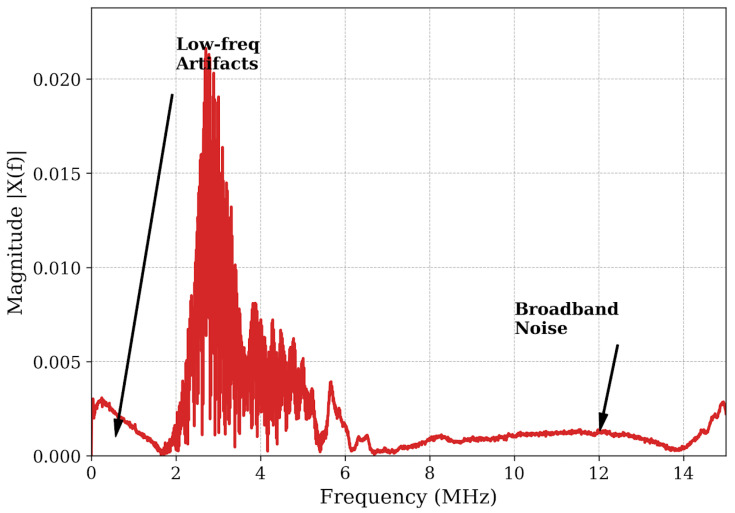
Frequency spectrum of the raw signal.

The resulting spectrum in Figure 4 reveals a critical physical phenomenon: although the transducer operates at a nominal center frequency of 4 MHz, the received energy is predominantly concentrated around 2.71 MHz. This significant frequency downshift of ~1.3 MHz indicates a strong filtering effect imposed by the material microstructure. According to recent studies on ultrasonic attenuation in polycrystalline steel, higher-frequency components experience accelerated attenuation due to grain scattering (α~*f*^4^) [24], causing the spectral centroid to shift toward lower frequencies. To quantitatively verify that material attenuation is responsible for this significant downshift, an estimation based on the spectral centroid downshift method was conducted. As demonstrated in recent studies [27,28], for a broadband pulse propagating through a dispersive medium, a mathematical relationship exists linking the shift in center frequency (Δ*f*) to the attenuation coefficient slope (α), the spectral variance of the transducer (σ^2^), and the propagation distance (*x*), expressed as Δ*f* = x·σ^2^·α [27]. Furthermore, Xu et al. [29] recently confirmed that this centroid downshift is highly pronounced in polycrystalline metals due to severe high-frequency grain boundary scattering. For the representative 8.0 mm defect (*x* = 1.6 cm round-trip) interrogated by the highly damped pulse (≈150 ns width, yielding a bandwidth σ ≈1.5 MHz), the 1.29 MHz downshift yields an estimated material attenuation slope of approximately 3.11 dB/(cm.MHz). This calculated value falls precisely within the established empirical range for high-carbon polycrystalline steels (1 to 4 dB/(cm.MHz)) [24]. Thus, the observed downshift is a physically expected consequence of the material’s inherent frequency-dependent acoustic absorption. Figure 4 also highlights distinct noise components: high-energy low-frequency artifacts (<1 MHz) arising from mechanical coupling instability, and broadband electronic noise (>10 MHz).

Based on this spectral insight, a 4th-order Butterworth band-pass filter was designed due to its maximally flat passband response, which is vital for preventing phase distortion and preserving the temporal integrity of the pulse. A critical design decision was the selection of the passband from 0.5 MHz to 15 MHz. The lower cutoff of 0.5 MHz was explicitly chosen to effectively eliminate macro-mechanical artifacts and low-frequency vibrational noise, which are highly prevalent in practical railway field environments. Crucially, the upper cutoff was intentionally extended to 15 MHz (≈3.75 f_nominal_) rather than a narrower limit. This wide-band strategy is essential for time-domain metrology; as demonstrated in recent signal processing literature [25], excessively narrow filtering restricts the signal bandwidth, causing pulse broadening and time-domain ringing, which degrades temporal resolution. By maintaining a wide upper stopband, we preserve the high-frequency harmonic components required to maintain a sharp rising edge.

**Figure 5:**
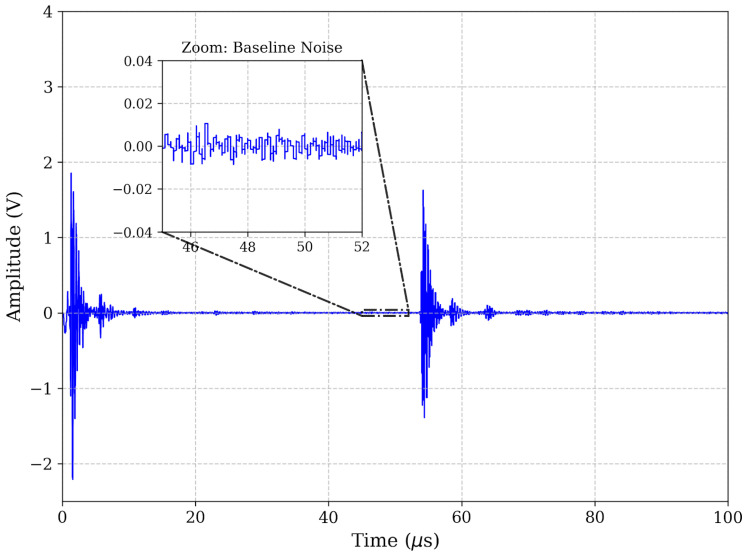
Signal after band-pass filtering in time domain.

**Figure 6:**
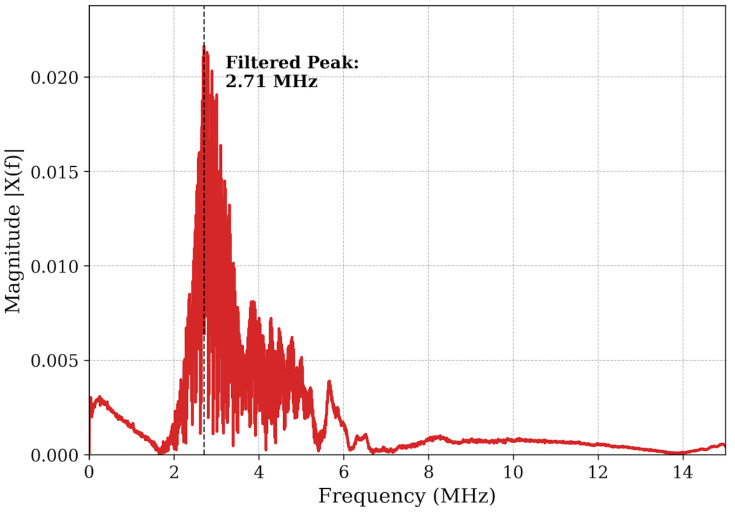
Frequency spectrum after band-pass filtering (0.5–15 MHz).

The result of this filtering strategy is shown in Figure 5 (time-domain) and Figure 6 (spectrum). The filtered signal exhibits a pristine baseline and a clearly defined echo. To explicitly visualize the improvement in the *SNR* achieved by the filter, zoomed-in insets focusing specifically on the baseline noise floor (45–52 μs) have been incorporated into the time-domain plots. As demonstrated in these close-up views (scaled to ±0.04 V), the raw signal as shown in Figure 3 suffers from noticeable low-frequency baseline wander and high-frequency jaggedness. The band-pass filter effectively flattens this baseline to near-zero amplitude prior to the echo arrival, yielding a highly pristine waveform as shown in Figure 5. This significant noise reduction is critical for the subsequent phase-independent envelope extraction. To further eliminate phase ambiguity—a common source of “cycle-skipping” errors—the instantaneous energy envelope was extracted using the Hilbert Transform. The analytic signal *z*(t) is constructed from the filtered signal *s_filtered_(t)* and its Hilbert Transform *H*[.]:

(2) $$z\left(t\right)=\;s_{filtered}\left(t\right)+\;jH\left[s_{filtered}\left(t\right)\right]$$

Physically, Equation (2) constructs a complex-valued signal where the imaginary part is a 90° phase-shifted version of the real signal. This allows for the calculation of the instantaneous energy envelope *E*(t), defined as the modulus of the analytic signal:

(3) $$E\left(t\right)=|z(t)|=\sqrt{{{(s}_{filtered}\left(t\right))}^{2}+{\left(H\left[s_{filtered}\left(t\right)\right]\right)}^{2}}$$

**Figure 7:**
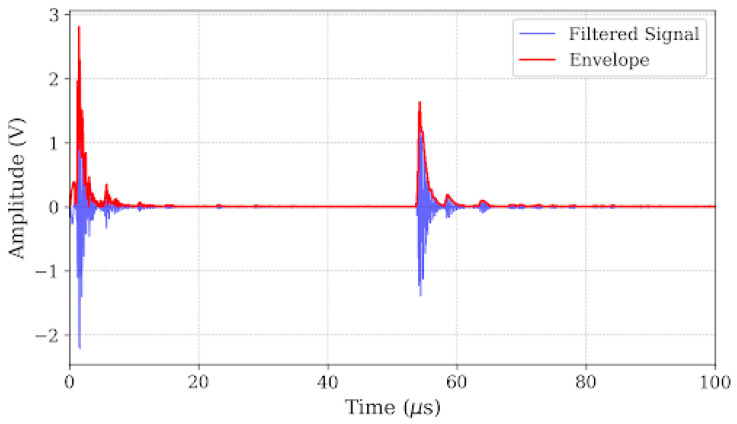
Comparison of filtered signal and its envelope.

**Figure 8:**
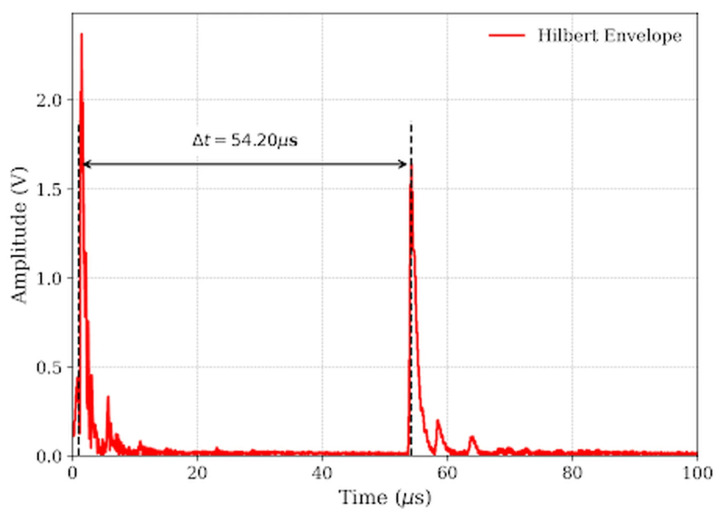
Signal envelope extracted via Hilbert Transform.

The significance of Equation (3) lies in its ability to decouple the amplitude information from the phase information. Unlike raw waveforms where the peak position depends on the oscillating carrier phase (which can shift due to dispersion), the envelope *E*(t) represents the total energy distribution of the wave packet. As illustrated in Figure 7 and Figure 8, this envelope provides a smooth, unipolar curve where the peak aligns with the energy centroid. This approach allows for a robust determination of the Δ*t* = 54.20 μs for the 8 mm defect, significantly reducing timing jitter compared to zero-crossing methods [26].

### 3.2. In Situ Velocity Calibration and Linearity Analysis

A prevalent source of systematic error in ultrasonic crack sizing is the ambiguity of the propagation velocity. Conventional models often simplify the problem by assuming the speed of sound in free air *c*_air_ ≈ 343 m/s. However, this assumption neglects the confinement effects where the crack width is comparable to the acoustic wavelength.

To address this, an in situ calibration was conducted. It is important to note that while the artificial defects ranged from 0.1 mm to 10.0 mm, the 0.1 mm defect was excluded from the quantitative regression analysis. At this extremely shallow depth, the defect echo returns almost simultaneously with the excitation pulse. Given the system’s pulse width of approximately 150 ns and the transducer’s electromechanical recovery time, defects shallower than 0.2 mm fall within the “dead zone” (or blind zone) where the receiver is saturated by the initial main bang. Consequently, the reflected signal from the 0.1 mm crack is masked by the excitation tail, making accurate ToF extraction unfeasible without specialized delay-line transducers.

**Figure 9:**
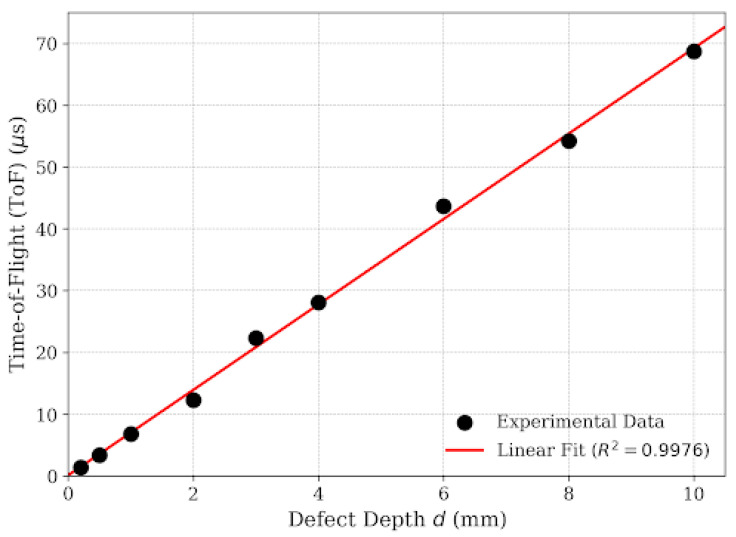
Linear regression of Time-of-Flight vs. defect depth.

Therefore, the calibration was performed for the valid range of 0.2 mm to 10.0 mm. Figure 9 presents the linear regression analysis of the measured *ToF* versus actual crack depths for this measurable range. The data demonstrates an exceptional linear correlation (R^2^ ≈ 0.9976), governed by the empirical model:

(4) $$ToF\left(\mu s\right)=6.9141d+0.0970$$

From the slope of this regression line (*k* = 6.9141 μs/mm), the effective propagation velocity *v_eff_* inside the crack is derived:

(5) $$v_{eff}=\frac{2}{k}10^{3}=\frac{2000}{6.9141}=289.3\;m/s$$

This experimentally determined velocity of 289.3 m/s is approximately 15.6% lower than the standard speed of sound in air. This retardation effect is consistent with the theory of sound propagation in narrow tubes/slits, where viscous drag significantly reduces the phase velocity. This confinement effect is physically governed by viscous drag. In narrow geometries, the friction between the oscillating air particles and the rigid steel boundaries creates an acoustic boundary layer that retards the phase velocity, resulting in the observed 15.6% reduction. While the spectral attenuation and frequency downshift are primarily governed by propagation through the bulk rail steel, the velocity retardation is a boundary effect occurring strictly within the air-filled confinement of the crack gap.

To quantify the impact of this deviation, consider the representative case of the 8 mm crack, which exhibited a measured ToF of Δt = 54.20 μs (Figure 8). If the standard speed of sound in air *c_air_* =343 m/s were substituted into the depth equation, the calculated depth would be as follows [8]:

(6) $$d_{calc}=\frac{1}{2}c_{air}\Delta t=9.3\;mm$$

Compared to the actual depth of 8.00 mm, this assumption leads to an overestimation of 1.30 mm, corresponding to a relative error of 16.25%. By calibrating for *v*_eff_, our system eliminates this systematic bias, ensuring sub-millimeter accuracy.

### 3.3. Optimization of Excitation Parameters and System Design Implications

While the advanced signal processing algorithms and the calibrated velocity model provide the theoretical foundation for high-accuracy measurement, the ultimate performance of the ultrasonic inspection system is physically constrained by the quality of the excitation source. In practical instrumentation design, particularly for portable rail inspection devices, there is always a trade-off between power consumption, circuit complexity, and measurement fidelity. To establish an optimized hardware configuration, this study systematically analyzes the impact of excitation voltage and pulse width on the measurement relative error. To generate the parameter optimization data, the baseline experimental setup (utilizing the 8.0 mm defect) was maintained. We systematically varied the pulser configuration, sweeping the excitation voltage from 60 V to 180 V and the pulse width from 50 ns to 1100 ns, while continuously recording the corresponding ToF errors.

**Figure 10:**
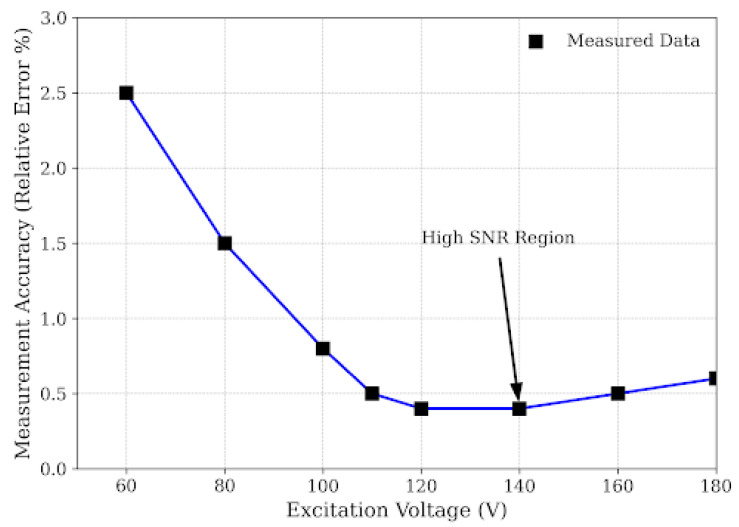
Effect of excitation voltage on measurement accuracy.

The relationship between the excitation voltage (*V_exc_*) and measurement accuracy, presented in Figure 10, reveals a distinct non-linear behavior characterized by two operating regimes. In the noise-limited regime where *V_exc_* < 80 V, the measurement error is significantly high (>2.5%) and exhibits large variance. From a physical perspective, ultrasonic attenuation in polycrystalline steel is severe due to grain boundary scattering. When the excitation energy is insufficient, the amplitude of the returning echo from small cracks falls closer to the thermal noise floor of the receiver amplifier. Under these low-SNR conditions, the Hilbert envelope becomes jagged, causing the peak detection algorithm to lock onto noise spikes rather than the true defect echo. This observation aligns with the findings of Bi et al. [24], who reported that in highly attenuative materials, a minimum acoustic pressure threshold is required to overcome scattering losses. Conversely, as the voltage increases beyond 110 V, the relative error drops sharply and stabilizes below 0.5%. The “knee” of this curve at ≈110 V represents the optimal operating point where the echo amplitude provides sufficient *SNR* (>20 dB) for robust envelope extraction without inducing non-linear saturation in the receiver pre-amplifier.

**Figure 11:**
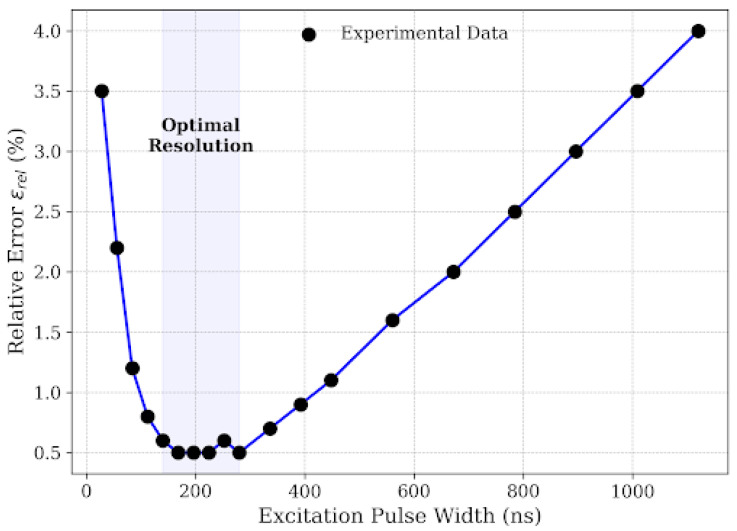
Optimization of pulse width for minimal error.

This voltage dependency has critical implications for system design. Many low-cost commercial ultrasonic modules operate at standard logic voltages (5 V–12 V). Our results prove that such low-voltage excitation is fundamentally unsuitable for rail steel inspection due to the material’s high attenuation coefficient. Consequently, the design of a specialized rail crack detector necessitates a dedicated high-voltage step-up circuit capable of generating ≥ 110 V pulses. While this increases circuit complexity compared to low-voltage solutions, Figure 10 serves as empirical evidence that this power trade-off is non-negotiable for achieving sub-millimeter accuracy. Complementing the voltage analysis, Figure 11 investigates the impact of excitation pulse width (*t*_pw_) on measurement accuracy, revealing a classic engineering trade-off between sensitivity and axial resolution. At very narrow pulse widths *t*_pw_ < 100 ns, the error is high because the pulse duration is shorter than the piezoelectric transducer’s electrical time constant, preventing full mechanical displacement and resulting in a weak acoustic emission. On the other hand, extending the pulse width beyond 300 ns leads to a degradation in accuracy due to bandwidth narrowing and the extension of the “dead zone.” A longer pulse corresponds to a narrower main lobe in the frequency domain, which reduces the effective bandwidth and causes “smearing” of the echo in the time domain [25]. Furthermore, wider pulses extend the ring-down time of the transducer, causing the tail of the excitation pulse to mask the returning echoes from shallow cracks (e.g., <2 mm).

The experimental data identifies an optimal operating window of 140 ns–280 ns, centered around 150 ns. This value closely matches the half-period of the resonant frequency (T/2 ≈ 143 ns), which is theoretically the most efficient duration to excite a resonant system. Unlike complex coded excitation sequences suggested in recent air-coupled studies by Zhang et al. [26], our results indicate that for contact-based rail inspection, a simple square pulse tuned to this optimal window provides a superior balance of signal strength and temporal resolution while remaining computationally efficient for embedded microcontrollers. Collectively, these findings provide a clear design guideline: to achieve reliable rail crack sizing, the ultrasonic instrument must implement a high-voltage pulser (>110 V) with a tunable pulse width fixed near 150 ns, prioritizing burst-mode energy management to accommodate the high power requirements.

### 3.4. Comparison with Related Works

To position the contributions of this study within the broader context of rail inspection technology, a comparative analysis was conducted against relevant recent studies. Table 1 summarizes the key methodological differences between our proposed framework and other state-of-the-art ultrasonic approaches.

While advanced non-contact methods such as Laser Ultrasonic Guided Waves utilized by Pathak et al. [9] and Lian et al. [11] offer the advantage of remote inspection, they inherently suffer from lower *SNR* and require complex, bulky optical instrumentation. Pathak’s work [9] effectively detects rail foot flaws but relies on dispersion curves that are computationally intensive to resolve for depth sizing. Similarly, the air-coupled approach by Bühling et al. [21] avoids coupling issues but assumes the *c*_air_, which, as demonstrated in our Section 3.2, introduces systematic errors (>15%) when applied to narrow crack geometries in steel due to confinement effects.

In terms of signal processing, recent works have proposed sophisticated algorithms to improve ToF estimation. Yang et al. [16] utilized a defect peak tracking model, and Tumšys [22] proposed a zero-crossing method based on filtered signals. While effective for laboratory specimens, zero-crossing methods remain sensitive to phase distortions caused by dispersive attenuation in rail steel. Our adoption of the Hilbert envelope, similar to the robust approach recently validated by Yu and Kim [25] for ultrasonic flowmeters, provides a phase-independent estimation of energy arrival. Our work extends this efficacy from fluid dynamics to solid mechanics by coupling it with a physically calibrated *v*_eff_.

Regarding excitation strategies, Wang et al. [26] recently demonstrated the benefits of “Composite Pulse Excitation” (coded waveforms) to enhance SNR in highly attenuative materials like wood. While scientifically rigorous, coded excitation requires complex correlation processing hardware (typically FPGA). In contrast, our hardware optimization analysis (Section 3.3) proves that for rail steel, a simpler High-Voltage (>110 V), Tuned-Width (~150 ns) Square Pulse is sufficient to achieve sub-millimeter accuracy (R^2^ ≈ 0.9976). This finding is significant for reducing the cost and complexity of portable rail inspection devices without compromising performance.

Ultimately, the distinguishing feature of this study is the integration of the *v*_eff_ model. Unlike theoretical works [10] or standard ToF applications that rely on handbook velocity values, our method experimentally derives *v*_eff_ ≈ 289.3 m/s. This implicitly compensates for the viscous boundary layer losses inside the crack, resulting in superior sizing accuracy.

## 4. Conclusions

This study validates that integrating effective velocity calibration with Hilbert envelope extraction is decisive for enhancing ultrasonic crack sizing accuracy in railway rails. By addressing the observed spectral downshift to 2.71 MHz, the implementation of Hilbert Transform envelope extraction provided a robust, phase-independent ToF determination mechanism, effectively overcoming the limitations of broadband noise and dispersion inherent in standard zero-crossing methods. Crucially, the investigation quantified the pivotal role of propagation velocity, demonstrating that the effective velocity within narrow surface cracks (*v*_eff_ ≈ 289.3 m/s) deviates by approximately 15.6% from the free-space sound speed due to boundary layer confinement effects; incorporating this calibrated parameter into the depth estimation model resulted in exceptional linearity (R^2^ ≈ 0.9976) for defects ranging from 0.2 mm to 10.0 mm. Furthermore, the study established that optimizing excitation parameters—specifically employing high voltage (≥110 V) and a tuned pulse width (≈150 ns)—is essential to maximize the efficacy of these signal processing and calibration techniques, thereby offering a validated framework for high-precision, portable rail inspection systems. Future research will extend this calibrated effective velocity model to address the complex morphologies of real-world rail defects, including tightly closed and angled rolling contact fatigue cracks.

## Figures and Tables

**Table 1:** Comparative analysis of the proposed method with existing ultrasonic techniques.

Reference	Method/Technique	Wave Mode	Velocity Model	Key Advantage	Limitation/Gap Addressed by this Work
Pathak et al. [9]	Laser Ultrasonic Guided Wave	Guided Waves	Dispersion Curves	Detects rail foot flaws; Non-contact	Complex optical setup; Dispersion analysis is computationally heavy.
Katz et al. [10]	Theoretical Modelling	Bulk Waves	Theoretical Elasticity	Predicting beam trajectory	Purely analytical; Lacks experimental calibration for real-world attenuation.
Lian et al. [11]	Laser Ultrasonic (VMD)	Rayleigh Surface	Standard Surface Wave	High resolution for V-cracks	High cost; Low SNR compared to contact methods.
Yang et al. [16]	Peak Tracking Model	Bulk Waves	Standard	Auto-diagnosis of signal peaks	Computational complexity; Does not address “effective velocity” shift.
Bühling et al. [21]	Air-Coupled ToF	Longitudinal	Speed of Sound in Air	Robustness in air transmission	Assumes *c*_air_ which causes significant errors in narrow steel cracks.
Tumšys [22]	Zero-Crossing (Filtered)	Lamb Waves	Phase/Group Velocity	Improved center freq estimation	Zero-crossing is sensitive to phase jitter; Focused on composite plates.
Yu & Kim [25]	Peak Detector Envelope	Ultrasonic Pulse	Flow Velocity	Low-complexity hardware	Domain is fluids; Our work adapts this robustness to solid rail steel.
Zhang et al. [26]	Composite Pulse Excitation	Longitudinal	Standard	High SNR in attenuative media	Requires complex coded excitation; Our work proves Simple Tuned Pulse is sufficient.
This Work	Optimized DSP and *v*_eff_ Calibration	Pulse-Echo Bulk	Effective Velocity	High Linearity (R^2^ ≈ 0.9976); Portable Design.	Addresses velocity ambiguity (*v*_eff_); Optimized for embedded systems.

## Data Availability

The datasets generated and/or analyzed during the current study are available from the corresponding author on reasonable request.

## Abbreviations

The following abbreviations are used in this manuscript:

*NDE*	Non-destructive evaluation
*ToF*	Time of flight
*NDT*	Non-destructive testing
*SNR*	Signal-to-noise ratio
*c*_air_	Speed of sound in air
*DSP*	Digital signal processing
*d*	Defect depth
*s*_raw_	Raw time-domain signal
FFT	Fast Fourier Transform
Δ*f*	Shift in center frequency
α	Attenuation coefficient slope
σ^2^	Spectral variance of transducer
*x*	Propagation distance
*v*_eff_	Effective propagation velocity
*V_exc_*	Excitation voltage
*t*_pw_	Pulse width
Δ*t*	Time interval between the transmission pulse and this reflected echo
*ε*_rel_	Relative error
